# Supplementary material for: Evaluating case management as a complex intervention: Lessons for the future
Source: PLoS One. 2019 Oct 31;14(10):e0224286. doi: 10.1371/journal.pone.0224286 (PMC6822731; doi:10.1371/journal.pone.0224286)
Supplement: S1 Table — (DOCX) [file pone.0224286.s001.docx]

**S1 Table.** Description of the number of patients by CMI category and by disability profile, and description of the number of projects, by CMI category.

|  | IADL (cogn.) | Func., cogn. | Func., cogn., behav. | Total | Total |
| --- | --- | --- | --- | --- | --- |
|  | N patients | N patients | N patients | N patients | N projects |
| **basic care coordination** | 219 | 81 | 24 | 324 | 7 |
| **low-intensity CMI** | 404 | 349 | 106 | 859 | 16 |
| **high-intensity CMI** | 1307 | 410 | 116 | 1833 | 27 |
| **Total** | 1930 | 840 | 246 | 3016 | 40 |
