# Supplementary material for: Evaluating case management as a complex intervention: Lessons for the future
Source: PLoS One. 2019 Oct 31;14(10):e0224286. doi: 10.1371/journal.pone.0224286 (PMC6822731; doi:10.1371/journal.pone.0224286)
Supplement: S2 Table — (DOCX) [file pone.0224286.s002.docx]

**S2 Table.** Evaluation of balance of the BelRAI covariables between study arms, by disability profile, for the total sample of “Protocol 3.”

|  | IADL (cogn.) | | | Func., cogn. | | | Func., cogn., behav. | | |
| --- | --- | --- | --- | --- | --- | --- | --- | --- | --- |
|  | W | NC | C | W | NC | C | W | NC | C |
| **ADL** | | | | | | | | | |
| smd | 0.09 | 0.01 | 0.20 | 0.35 | 0.16 | 0.15 | 1.72 | 0.35 | 0.34 |
| VR | 1.88 | 1.64 | 1.03 | 2.67 | 1.15 | 1.43 | - | 1.68 | 1.37 |
| **IADL** | | | | | | | | | |
| smd | 0.30 | 0.05 | 0.29 | 0.36 | 0.01 | 0.05 | 1.26 | 0.35 | 0.51 |
| VR | 1.88 | 2.18 | 4.67 | 1.04 | 1 | 1.13 | - | 1 | 1.61 |
| **CPS** | | | | | | | | | |
| smd | 0.26 | 0 | 0.13 | 0.49 | 0.05 | 0.12 | 0.80 | 0.14 | 0.74 |
| VR | 1.84 | 1.47 | 1.59 | 1.50 | 1.07 | 1.18 | - | 2.21 | 2.01 |
| **DRS** | | | | | | | | | |
| smd | 0.27 | 0.16 | 0.20 | 0.44 | 0.20 | 0.10 | 1.44 | 0.14 | 0.08 |
| VR | 1.26 | 1.13 | 1.69 | 5.86 | 1.24 | 1.43 | - | 1.80 | 1.12 |
| **Behav** | | | | | | | | | |
| smd | 0.32 | 0.12 | 0.06 | 0.60 | 0.26 | 0.24 | 0.66 | 0.13 | 0.01 |
| VR | 2.36 | 2.56 | 1.23 | 6.47 | 3.64 | 2.72 | - | 1.29 | 1.26 |
| **Average Standardized Mean Difference** | | | | | | | | | |
|  | 0.24 | 0.11 | 0.21 | 0.44 | 0.13 | 0.15 | 1.02 | 0.26 | 0.32 |
| **Geometric Mean Variance Ratio** | | | | | | | | | |
|  | 1.74 | 1.66 | 1.60 | 2.87 | 1.44 | 1.42 | - | 1.54 | 1.38 |
| **Treated** | | | | | | | | | |
| N | 267 | 1135 | 530 | 121 | 919 | 1276 | 29 | 214 | 431 |
| **Control** | | | | | | | | | |
| N | 30 | 70 | 49 | 9 | 54 | 144 | 1 | 10 | 24 |

The evaluation of the balance of the BelRAI covariables was done for each of the propensity score models, that is for each level of presence of an informal caregiver (W: without FC, NC: Non cohabitant FC, C: Cohabitant FC) within different disability categories. N: indicates the number of participants per study arm.
