# Supplementary material for: Evaluating case management as a complex intervention: Lessons for the future
Source: PLoS One. 2019 Oct 31;14(10):e0224286. doi: 10.1371/journal.pone.0224286 (PMC6822731; doi:10.1371/journal.pone.0224286)
Supplement: Attachment — Submitted filename: Response to Reviewers.docx [file pone.0224286.s003.docx]

Response to reviewers

Dear reviewers and editor,

Thanks a lot for the smooth process of reviewing our article. Hereafter, you will find response to the two reviewers’ comments.

We remain, of course, ready to clarify further our work, would they be any other queries.

Warm regards,

Anne-Sophie Lambert

Reviewer #1: Dear authors, this manuscript tries to highlight a study but it was difficult reading it.
For example in line 69 the authors mentioned 50 Projects, but it was not explained which 50 Projects. Then in line 129 six case studies were mentioned. I lost the overview. How many patients were enrolled?
All in all I have to conclude that the manuscript is not worth for publication.

I would like first thank you for your pertinent comment. I totally agree with your comment. I include different elements in the paper to clarify the lines 69 and 129.

First in the introduction, I justified the number of projects (line 63 and 69). 75 projects were included in the Protocol 3 programme. Among them, 50 projects implemented Case Management. The others delivered psychological support, occupational therapy or night care. This paper focuses on the 50 projects that implemented Case Management.

Second in the methodology, I added precisions and references who justified the choice to analyze only six projects in-depth (line 113 - 125). The classification of CM projects started by the building of a normative grid: a list which captures what the stakeholders of the projects believed to be the key components of the projects [1]. To reach this objective, we used multiple embedded case study. This qualitative approach focuses on a small number of cases to highlight a causal relation assumed to be present in a large number of cases [2]. The cases are selected to extract as much diversity as possible regarding the projects‘ characteristics [2, 3]. Thus, we used six in-depth cases studies, selected among the 50 projects that implemented CM. These six projects were selected on the basis of (1) their diversity (i.e. the profile of the case manager (i.e. nurse, occupational therapist, psychologist or social worker)); (2) their geographical location (urban or rural); and (3) their collaboration with an existing organization that, among other criteria, coordinated care.

Third, in the first version of the manuscript, the number of patients enrolled was included in the S1 Appendix. However, I agree with you, it is necessary to include this information in the manuscript. So, I added the number of projects and the number of patients included in each CM category in the manuscript (lines 143, 145, 147). The total number of beneficiaries and the total number of controls were corrected (line 170). And the reference to S1 Appendix was added when I presented the number of beneficiaries per CM category (line 202, 203, 204).

Reviewer #2: This is a well conducted mixed methods study on a very innovative style and a comprehensive approach to an increasingly relevant problem of frail people. The methodology is described well and in detail.

 I would like thank you a lot for your positive comment.
